# Hypertrophic spinal pachymeningitis caused by ANCA-associated vasculitis revealed by ^18^F-FDG PET/CT: A case report

**DOI:** 10.1097/MD.0000000000024388

**Published:** 2021-01-22

**Authors:** Meiqi Wu, Jingyun Ren, Yaping Luo

**Affiliations:** a Department of Nuclear Medicine, Chinese Academy of Medical Sciences and Peking Union Medical College Hospital; b Beijing Key Laboratory of Molecular Targeted Diagnosis and Therapy in Nuclear Medicine; Beijing, PR China.

**Keywords:** ^18^F-FDG, ANCA-associated vasculitis, hypertrophic spinal pachymeningitis, PET/CT

## Abstract

**Rationale:**

Antineutrophil cytoplasmic antibody (ANCA)-associated vasculitis (AAV) can involve the central nervous system in estimatedly 15% of patients. Hypertrophic pachymeningitis causes inflammatory hypertrophy of the cranial or spinal dura mater and patients present with various neurological deficits. ANCA-associated hypertrophic spinal pachymeningitis has rarely been reported in literature. We report a case of AAV presenting with hypertrophic spinal pachymeningitis detected by ^18^F-FDG PET/CT.

**Patient concerns:**

A 66-year-old woman diagnosed with antineutrophil cytoplasmic antibody (ANCA)-associated vasculitis developed back pain, bilateral lower limb weakness, dysuria, and dysporia 1 month ago.

**Diagnosis:**

Contrast-enhanced MRI showed thickening and enhancement of the dura mater in the thoracic cord. Intraspinal hypermetabolism in the corresponding region was observed on ^18^F-FDG PET/CT. The patient was finally diagnosed with ANCA-associated hypertrophic spinal pachymeningitis.

**Interventions:**

The patient was treated with a higher dose of prednisone and cyclophosphamide.

**Outcomes:**

After 2-week treatment, the patient's neurological symptoms improved rapidly and laboratory findings were ameliorated. A repeated contrast-enhanced MRI showed partial improvement of the disease in the thoracic cord.

**Lessons:**

^18^F-FDG PET/CT and contrast-enhanced MRI can aid in the clinical diagnosis and surveillance in AAV-associated hypertrophic spinal pachymeningitis and potentially facilitate early recognition and intervention to prevent irreversible neurological impairment.

## 1 Introduction

Antineutrophil cytoplasmic antibody (ANCA)-associated vasculitis (AAV) is a clinical entity of necrotizing vasculitis predominantly affecting small vessels without deposition of immune complexes. It is usually associated with myeloperoxidase (MPO) ANCA or proteinase-3 ANCA.^[1]^ AAV patients typically present with constitutional symptoms besides specific organ symptoms, such as fatigue, weight loss, and fever.^[1]^ Various organs and systems can be involved, including ear/nose/throat, trachea, or lungs, kidney, skin, and nervous system.^[2]^ Central nervous system (CNS) involvement occurs in less than 15% of AAV patients, presenting as hypertrophic pachymeningitis, cerebrovascular events, hypophysitis or mass lesions.^[2–5]^

ANCA-associated hypertrophic pachymeningitis causes inflammatory hypertrophy of the dura mater and compression of the adjacent nerve and blood vessels, resulting in neurological deficits.^[6–8]^ It mostly affects cranial dura, leading to manifestations of headache and cranial neuropathies.^[6,9]^ AAV-associated hypertrophic spinal pachymeningitis, however, rarely occurs, with only several cases reported in literature.^[7,8,10–12]^ In this study, we report a case of AAV presenting with hypertrophic spinal pachymeningitis detected by ^18^F-FDG PET/CT.

## 2 Case presentation

A 66-year-old woman presented with intermittent fever (Tmax 38.5°C), myalgia, and malaise for 5 months. Laboratory examinations showed elevated serum level of MPO-ANCA (51 RU/ml), increased ESR (105 mm/hour) and hsCRP (19.91 mg). Pathogenic screening had no positive findings. AAV was suspected and the patient was treated with prednisone (40 mg/day with gradual tapering) and hydroxychloroquine (0.3 g/day). Her symptoms were relieved and serum MPO-ANCA decreased to undetectable level after 2-month treatment. ESR and hsCRP also decreased significantly. One month later, the patient developed chest pain and cough with elevated D-dimer. Computer tomography pulmonary angiography (CTPA) detected thrombus in the branch of right common basal artery, suggesting pulmonary embolism. She was subsequently treated with anticoagulation therapy with low molecular weight heparin and warfarin.

**Figure 1:**
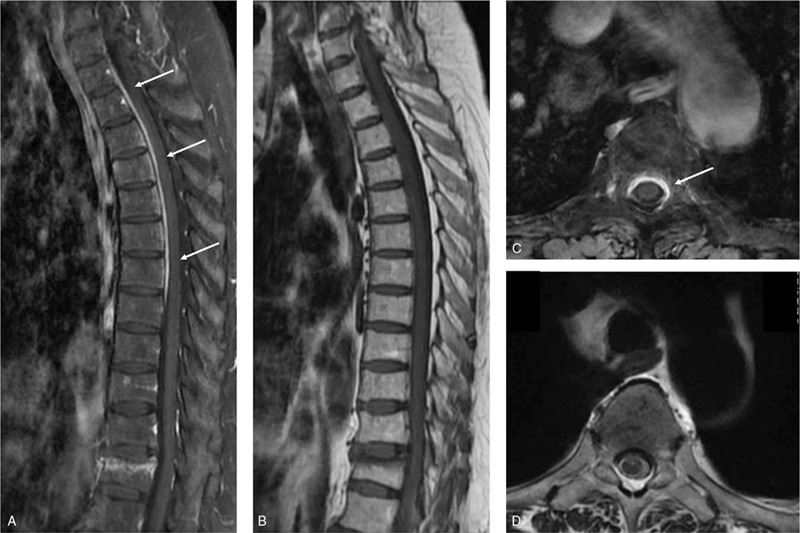
Contrast-enhanced MRI findings. (A) Sagittal MRI (T1WI+contrast-enhanced (CE)) of the spine showed diffuse thickening and enhancement of the frontal portion of the dura mater at upper to middle thoracic cord. (B) No obvious abnormality was noted on T2WI. (C,D) Axial MRI (C, T1WI+CE; D, T2WI) at the level of T4 showed changes at corresponding regions. Arrows: enhancement of the thoracic spinal dura mater.

One month ago, the patient gradually developed back pain, bilateral lower limb weakness, dysuria and dysporia, when on a dose of 5 mg prednisone per day. On admission, physical examination revealed lower limb weakness with positive Babinski's sign. Laboratory tests showed elevation of ESR (72 mm/hour) and hsCRP (64.36 mg/L), while MPO-ANCA was normal. Other serological antibodies, including antinuclear antibody, anti-double stranded DNA antibody, anticardiolipin antibody were also negative. Serum immunoglobulin (Ig) G, IgA, IgM, IgG4, and complement components were within normal ranges. Brain magnetic resonance imaging (MRI) was unremarkable but contrast-enhanced MRI of the spine showed diffuse thickening and enhancement of the dura mater at the level of 1st to 7th thoracic cord, without obvious abnormalities on T2-weighted image (T2WI Fig. 1).

**Figure 2:**
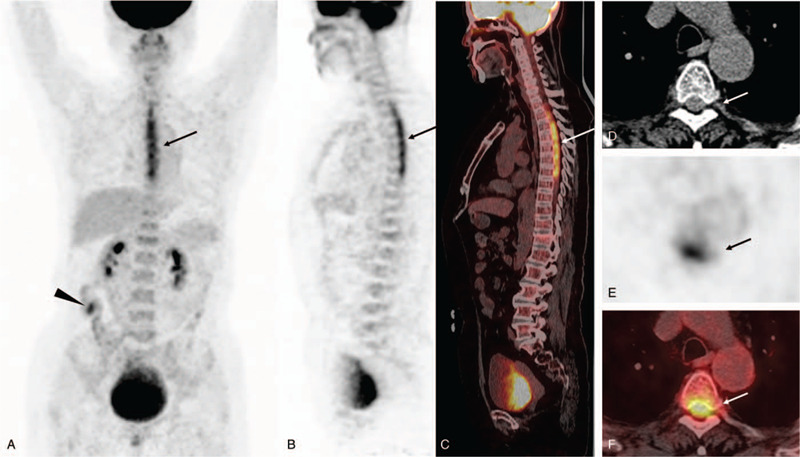
^18^F-FDG PET/CT findings. (A) MIP of FDG-PET showed intense hypermetabolism in the spinal cord (arrow) and an FDG-avid focus in the ascending colon (arrowhead); (B, C) Sagittal PET and fusion images showed increased FDG uptake in the spinal cord between the level of 1st and 7th thoracic vertebra (SUVmax 5.4, arrows); (D-F) Axial CT, PET and fusion images at the level of T4 showed diffuse intraspinal hypermetabolism (arrows).

Though AAV could attribute to pulmonary embolism and neurological disease, malignancy could not be excluded. Thus, the patient underwent ^18^F- fluorodeoxyglucose (FDG) Positron emission tomography/Computed tomography PET/CT (Fig. 2) for the underlying malignancy. It revealed increased ^18^F-FDG uptake in the spinal cord between the level of 1st and 7th thoracic vertebra (arrows), with the maximum of standardized uptake value (SUVmax) being 5.4. An FDG-avid focus in the ascending colon (arrowhead) was also noted, which was later proved to be a tubular adenoma by endoscopic polypectomy. No other hypermetabolic lesion was noted. With the findings on PET/CT, malignant myelopathy or inflammatory disease affecting intraspinal structures could not be differentiated.

Afterwards, cerebrospinal fluid (CSF) test showed normal CSF pressure, normal white blood cell count, and slightly increased protein (0.54 g/L). CSF interleukin-6 (23.0 pg/ml) and interleukin-8 (82.0 pg/ml) levels were increased. Tumor and paraneoplastic markers and examinations of pathogenic microorganisms (*Staphylococcus*, *Mycobacterium tuberculosis*, *Aspergillus*, *Treponema pallidum*, etc.) in serum and CSF were negative. Serum protein electrophoresis and immunofixation electrophoresis were normal. CSF cytological test showed lymphocytic inflammation without any sign of malignancy. Then the diagnosis of hypertrophic spinal pachymeningitis associated with AAV was established, and she was subsequently treated with a higher dose of prednisone (60 mg/day) and cyclophosphamide (0.4 g/week). By discharge after 2-week treatment, her neurological symptoms improved rapidly and laboratory findings were also ameliorated, with hsCRP (2.63 mg/L) and ESR (14 mm/hour) returned normal. A repeated contrast-enhanced MRI after 12 weeks also showed partial improvement of the disease in the thoracic cord compared with the prior MRI. The patient did not repeat spinal MRI in the following 1-year follow-up, but her neurological symptoms remained stable with gradual tapering or prednisone and she continued with remission-maintenance treatment of 5 mg prednisone and 100 mg azathioprine per day.

## 3 Discussion and conclusions

We have described a patient with suspected AAV who developed hypertrophic spinal pachymeningitis during the course of disease. Her hypertrophic spinal pachymeningitis presented as back pain, bilateral lower limb weakness, dysuria, and dysphoria. Contrast-enhanced MRI of the spine showed extensive dural inflammation of the thoracic spinal cord, and ^18^F-FDG PET/CT showed intraspinal hypermetabolism in the corresponding region indicating active inflammation in the spinal cord, leading to the diagnosis of hypertrophic spinal pachymeningitis.

Hypertrophic spinal pachymeningitis is an inflammatory disorder causing focal or diffuse thickening of the spinal dura mater. It usually presents as myelopathy and the clinical manifestations vary according to the involved segment of spinal dura. Thoracic dura involvement has been most frequently reported.^[8,10,12–20]^ The common symptoms include local pain, numbness, weakness of the limbs, difficulty in urination, and defecation. Etiologies of hypertrophic spinal pachymeningitis are various and could be generally summarized as autoimmune diseases, infections, and potentially malignancies. Pathological changes of AAV-associated hypertrophic spinal pachymeningitis are similar to other sites affected by AAV, characterized by fibrosis with infiltration of inflammatory cells, some accompanied by epithelioid granulomas or vasculitis; necrotizing granulomatous inflammation includes multinucleated giant cells, necrosis, and vasculitis in thickened dura mater; while inflammatory cell infiltration consists mainly of lymphocytes.^[6,9]^ Other autoimmune diseases such as IgG4-related disease, Sjogren disease and sarcoidosis may also cause hypertrophic spinal pachymeningitis;^[8,13,14,16]^ CNS infections such as syphilis and human T-lymphotropic virus type-1, heavy-chain disease^[20]^ are also reported to cause hypertrophic spinal pachymeningitis;^[16,18]^ besides, trauma could also result in inflammatory process of the spinal dura. Some patients found with hypertrophic spinal pachymeningitis without apparent causes are classified as idiopathic hypertrophic spinal pachymeningitis.^[9]^

In the present case, the etiology of hypertrophic spinal pachymeningitis was carefully differentiated with extensive workup:

1. CNS infection was unlikely with negative results of repeated examinations of pathogenic microorganisms in serum and CSF;
2. PET/CT, tumor markers, serum electrophoresis, and CSF cytological test did not show any evidence of solid tumor or hematological disease such as lymphoma or heavy-chain disease;
3. Other autoimmune diseases like IgG4-related disease or Sjogren disease were not considered due to normal serum level of IgG4 and negative antinuclear antibody.

Diagnosis of AAV in this patient is based on predominant sex, systemic symptoms, elevated MPO-ANCA and inflammation markers, remission of symptoms, and inflammation markers after treatment with glucocorticoids, besides exclusion of other factors that may cause such presentations, namely infections and neoplasms we mentioned above. Hypertrophic spinal pachymeningitis is not a common manifestation of AAV and the diagnosis of AAV-associated hypertrophic spinal pachymeningitis in this patient were established based on a suspected diagnosis of AAV and its close relation to the development of hypertrophic spinal pachymeningitis in the course of disease. The patient experienced typical neurological symptoms when on a small dose of prednisone, with elevation of ESR and CRP suggestive of reactivation of vasculitis. CSF examinations of elevated protein level, increased inflammatory markers like interleukin-6/8 in this patient reflected focal inflammatory process of the spinal meninges.^[7,21]^ Furthermore, good symptomatic, laboratory and radiological response to the treatment of glucocorticoids and immunosuppressants supported the final diagnosis of AAV-associated hypertrophic spinal pachymeningitis.

In patients suspected of AAV-associated hypertrophic spinal pachymeningitis, spinal contrast-enhanced MRI is an established essential modality for early diagnosis, therapy monitoring and recurrence surveillance. MRI findings of hypertrophic spinal pachymeningitis usually include diffuse or patchy thickening of the dura mater, most commonly involving thoracic cord, hypo- or iso-intensity on T1WI and hypo-intensity on T2WI, and can be markedly enhanced after contrast enhancement.^[6,8,10,12,19]^ Amelioration on MRI can be rather early after successful treatment with glucocorticoid and immunosuppressants.^[7,15,22]^ On the other hand, ^18^F-FDG PET/CT manifestations of AAV-associated hypertrophic spinal pachymeningitis have been rarely described. But from the few published cases, hypertrophic spinal pachymeningitis caused by AAV seems to share similar trait on ^18^F-FDG PET/CT with hypertrophic spinal pachymeningitis of other etiologies.^[11,23–25]^ On ^18^F-FDG PET/CT, hypertrophic spinal pachymeningitis usually present with diffuse or focal intraspinal hypermetabolism without prominent CT abnormalities, though in some cases, dural lesions appear as bulging masses.^[10,16]^ FDG avidity to hypertrophic spinal pachymeningitis is variable, from mild to high uptake.^[7,11,23–25]^ In addition to hypertrophic spinal pachymeningitis, intraspinal hypermetabolism on ^18^F-FDG PET/CT has also been reported in malignant and inflammatory diseases, including lymphoma, gliomas, meningeal carcinomatosis, neurosarcoidosis, infections, and other non-infectious inflammations.^[26–34]^ Therefore, the manifestations of hypertrophic spinal pachymeningitis in ^18^F-FDG PET/CT should be carefully differentiated with other diseases.

The gold standard of diagnosing AAV-associated hypertrophic spinal pachymeningitis is biopsy of dura mater, which may reveal fibrosis with infiltration of inflammatory cells, some accompanied by epithelioid granulomas or vasculitis.^[6,9]^ However biopsy of the dura is invasive and histological evidence might be difficult to obtain. CNS involvement is generally regarded as an organ-threatening manifestation in AAV, and timely treatment should be started even without pathological evidence.^[3,6]^ Remission-induction treatment with high-dose of glucocorticoids and cyclophosphamide or rituximab has been proven effective in CNS AAV.^[1,35]^ In patients suspected of AAV-associated hypertrophic spinal pachymeningitis, MRI and ^18^F-FDG PET/CT are of great value in clinical diagnosis and surveillance. PET/CT, together with contrast-enhanced MRI, may provide great value for early recognition and intervention of AAV-associated hypertrophic spinal pachymeningitis to facilitate a timely treatment and prevent irreversible neurological impairment.

## Author contributions

**Conceptualization:** Jingyun Ren, Yaping Luo.

**Data curation:** Meiqi Wu.

**Formal analysis:** Meiqi Wu.

**Funding acquisition:** Yaping Luo.

**Investigation:** Meiqi Wu.

**Validation:** Yaping Luo.

**Writing – original draft:** Meiqi Wu.

**Writing – review & editing:** Yaping Luo.
